# Drug-handling problems and expectations of the ideal pediatric drug—reported by children and their parents

**DOI:** 10.1007/s00431-022-04419-6

**Published:** 2022-02-23

**Authors:** Birthe Herziger, Sarah Jeschke, Ruth Melinda Müller, Martina Patrizia Neininger, Thilo Bertsche, Astrid Bertsche

**Affiliations:** 1 University Hospital for Children and Adolescents, Neuropaediatrics, Ernst-Heydemann-Straße 8, Rostock, 18057 Germany; 2 Clinical Pharmacy, Institute of Pharmacy, Medical Faculty, Leipzig University, Brüderstraße 32, Leipzig, 04103 Germany; 3 Drug Safety Center, Leipzig University and University Hospital, Brüderstraße 32, 04103 Leipzig, Germany

**Keywords:** Children, Parents, Drug administration, Drug administration routes, Drug-handling problems, Drug formulations

## Abstract

Experienced drug-handling problems and inadequately considered expectations for drug therapy have an unfavorable influence on therapy. We performed a questionnaire survey in (i) parents of 0–5-year-old children and (ii) 6–17-year olds and their parents. We assessed (A) experienced drug-handling problems and (B) expectations for drug therapy. (i) Forty-six parents and (ii) 103 children and their parents participated in the study. Experienced drug-handling problems were described by (i) 100% of parents and (ii) 62% of children and 70% of parents. Problems concerned with the preparation of the drug, dosing, compliance with the time interval, and acceptance. (i) Sixty-five percent of parents preferred a peroral route of drug administration, while (ii) 74% of children and 86% of parents did so. Preferred characteristics of peroral drug formulations, e.g., liquid versus solid drug formulations or flavor, were highly heterogeneous. Preferences of 6–17-year-old children and their parents matched in 43 to 66%.

*Conclusion*: Most children and their parents had already experienced drug-handling problems. Preferences concerning the ideal pediatric drug were highly heterogeneous and in about half of cases, preferences of children and their parents differed. Thus, the children should be approached directly. If information is solely gained from parents, the children’s needs might remain unmet.

**Table:** 

**What is Known:** *•* *Pediatric drug administration is complex and therefore error-prone.* *•* *Experiences and expectations of children and their parents should be considered.*	
**What is New:** *•Most pediatric patients and their parents have already experienced drug-handling problems.* *•* *Expectations concerning the ideal pediatric drug are highly heterogeneous. Parents are often insufficiently aware of those expectations in their children.*

**Supplementary information:**

The online version contains supplementary material available at 10.1007/s00431-022-04419-6.

## Introduction

Drug administration errors and drug-handling problems are common in children and adolescents [1–3]. For instance, a systematic review found a median prevalence of 16.3% administration errors among hospitalized children in the UK; errors in administration technique represented 53% of these errors [3]. In a multicenter study in four French-speaking countries, inappropriate administration accounted for the largest proportion of drug-related problems (29%) [1]. Dosing problems were the most frequently reported drug-related problems in a study from the UK and Saudi Arabia (67% of drug-related problems) [2].

Drug-handling problems are particularly relevant in children and adolescents because dosages and dosage forms appropriate and approved for children are frequently not available [4, 5]. Therefore, dosage forms developed for adults need to be adapted for children. Examples are the crushing or splitting of tablets. Furthermore, dosage forms that are particularly appropriate for children, such as liquid formulations, tend to require more process steps in preparation than the comparatively simple handling of tablets for adults.

As described in the framework for factors contributing to medication adherence by Peh et al. [6] medication-related factors have a key role for the patient’s adherence and, consequently, therapeutic success. Examples for relevant medication-related factors especially in pediatric patients are dosing, administration, the complexity of the medication regimen, or medication properties such as drug formulation. In addition, behavioral factors such as skills in the administration routine have been identified as relevant factors for medication adherence. However, it is still unclear which problems children and parents encounter in pediatric drug administration in routine care. We hypothesized that children themselves and parents can provide valuable insights from their experiences and expectations for the development and design of drug formulations to ease drug administration to children. For the development of future characteristics of dosage forms, we consider two aspects to be particularly relevant: on the one hand, past experiences with medication errors that significantly also shape future behavior [7], and on the other hand, expectations which can also take into account new aspects or aspects not experienced by the patient’s oneself.

So far, however, only a small number of studies addressed the perspective of children themselves and their parents on drug-handling problems. For instance, in a questionnaire survey performed in 2015 among children in five European countries, bad taste of medicines, pain during administration, and difficulties in remembering to take drugs were the most commonly reported problems that may prevent appropriate drug use [8]. For this reason, we performed a questionnaire survey of children and parents addressing (A) their own experiences in pediatric drug administration and (B) their expectations of an ideal pediatric drug.

## Materials and methods

### Setting and patients

After approval from the local ethics committee, we performed this prospective observational study at a German university hospital for children and adolescents from May 2019 to July 2020. We consecutively invited (i) parents of in- and outpatients aged 0 to 5 years and (ii) patients aged 6 to 17 years and their parents to take part in the questionnaire survey. During an inpatient stay or an appointment at the outpatient department, we approached patients and parents/caregivers who were legal guardians of the children. Requirements to participate in the study were in- or outpatient treatment, sufficient knowledge of the German language, and intellectual capacity to understand and answer the questions. Participation in this study was voluntary and without any compensation. Informed consent was gained from all participants. For the children’s participation, parents/legal guardians as well as children gave their consent.

### Questionnaire

An expert panel consisting of pharmacists, a neuropediatrician, and a child and adolescent psychotherapist developed a questionnaire (English translation of the original German questionnaire see Supplement 1). The questionnaire consisted mainly of closed questions with categorical answers the participants could choose from. The following key issues were addressed in the questionnaire from the perspective of children and parents:

(Part A) Experiences of drug-handling problems

(Part B) Expectations of the ideal pediatric drug

(B.1) Favored route of drug administration

(B.2) Favored characteristics of peroral drugs

(B.3) Description of the ideal pediatric drug in their own words

For the domain experiences, the questionnaire reflected common categories of drug-handling problems. We derived those categories from the European Medicines Agency’s *Guideline on pharmaceutical development of medicines for pediatric use* (EMA/CHMP/QWP/805880/2012 Rev. 2, 2013): experienced problems in drug preparation, problems concerning correct dosing, problems in keeping the time interval in everyday life, and acceptance problems of the child. Those questions resulted in the categories yes/no/do not know, and were thus measurable as frequencies. We were not only interested in those superordinate categories but also in the concrete problems the participants had experienced, because those experiences can be very valuable for the development of new dosage forms. Thus, participants who ticked yes for a specific question were asked to provide details in their own words. In the expectation domain, we were interested in finding out the most important characteristic of a drug for children according to the participants. As flavor is a major factor for the child’s acceptance, we have included a deepening question on this topic. We also included questions on the favored route of drug administration because the route of administration is also a major factor for the child’s acceptance. For these questions, we provided answers reflecting the most common drug characteristics but the participants had also the possibility to provide their own opinion. As those data were categorical, we can provide frequencies of the chosen answers. Multiple answers were not intended, but if participants did not feel able to choose only one answer, all chosen answers were considered for the evaluation. The last question was aimed at the free imagination of what the participants would want for the ideal drug. Therefore, we did not provide any specifications for this question.

Answers to open-ended questions were categorized by an expert panel consisting of pharmacists, a neuropediatrician, and a child and adolescent psychotherapist. The categories were derived from the participants’ answers. As we intended to present the answers as close as possible to the actual participants’ answers, we only clustered into superordinate categories such as “answer refers to liquid dosage form” or “answer refers to solid dosage form”, and present examples for participants’ answers. For the categorization, each member of the expert panel assigned the answers to the categories. If there was disagreement between the panel members concerning the chosen categories, a discussion and clarification was performed by the panel members to reach consensus. In part B, only one question required an open answer. As those answers were very heterogeneous, we refrained from clustering and decided to present a broad range of examples in the manuscript in order to reflect the participants’ perspectives.

To increase comprehensibility, the questionnaire contained photos and pictograms of drug formulations. We did not test validity but took quality assurance measures such as pre-testing with children and parents to improve readability, comprehensibility, clarity, completeness, and practicability of the questionnaire.

### Data assessment

The participants filled in a self-completion questionnaire that was handed out to parents and children after they agreed to participate in this study. Children aged 6–17 years and their parents each received their own questionnaires and completed them without mutual influence, e.g., the parent completed the questionnaire in the waiting area while the child had an examination such as an electroencephalography. When the children completed the questionnaire, the parents were asked not to influence their children in answering the questions. While they filled in the questionnaire, a member of the study team (always the same pharmacist) was present to assist in case participants had problems filling in the questionnaire. This applied especially to younger children who had difficulties in reading and/or writing. The member of the study team was instructed not to interfere to avoid an influence on the participants’ answers. This ensured a highly standardized data collection process for all participants.

#### Statistics

Statistical analysis was conducted by using Microsoft Excel 2019 (version 16.0, Microsoft Corporation, Redmond, WA, USA) and SPSS (version 21, IBM Corporation, Armonk, NY, USA). Frequencies are reported in absolute and relative numbers. Continuous data were described as median with first (25%) and third (75%) quartile (Q25/Q75) and minimum/maximum (min/max). We compared children’s and parents’ preferences concerning route of drug administration and favored characteristics of peroral drugs. For comparisons, we performed Kruskal–Wallis tests and Mann–Whitney *U* tests as appropriate. For these statistical tests, we excluded the data of those participants who gave multiple answers to single-choice questions. An adjusted *p*-value ≤ 0.05 was considered to indicate significance. For each in the parents’ group and the children’s group, we further performed univariate and multivariate binary logistic regressions to identify predictors for experienced problems or preferred drug characteristics. In the parents’ group, the set of independent variables consisted of the child’s age, child’s sex, intake of long-term medication by the child, intake of long-term medication by the parents, parents’ level of professional education, and whether the parents had a medical/pharmaceutical/nursing profession. In the children’s group, the set of independent variables consisted of the child’s age, the child’s sex, intake of long-term medication by the child, and whether the child attended a school for children with special needs. To increase readability, we only present those results significant in the univariate or multivariate setting. As the variables with significance in the multivariate setting were also significant in the univariate setting, we report *p*-values only for the multivariate setting in those cases.

## Results

### Patient characteristics

We included (i) 46 parents of children aged 0–5 years and (ii) 103 pediatric patients aged 6–17 years and their parents. For characteristics of the participants, see Table 1. The children’s diagnoses comprised a wide spectrum of disorders, among others epilepsy, headache/migraine, diabetes mellitus type 1, acute and chronic respiratory disorders, gastroenteritis, and urinary tract infections. For details on the children’s long-term medications, see Table 2.

**Table 1:** Characteristics of the participants

	**Children aged 0–5 years (*n* = 46)**	**Parents of children aged 0–5 years (*n* = 46)**	**Children aged 6–17 years (*n* = 103)**	**Parents of children aged 6–17 years (*n* = 103)**
Age (years)				
Median	2	33	12	41
Q25/Q75	1/3	30/37	9/15	37/45
Min./max	0/5	20/44	6/17	25/63
Sex (*n* (%))				
Female	13 (28)	37 (80)	48 (47)	89 (86)
Professional education of parents (*n* (%))				
Vocational		28 (61)		73 (71)
University degree		13 (28)		24 (23)
No degree		5 (11)		1 (1)
No statement		0 (0)		5 (5)
Profession of parents (*n* (%))				
Medical/pharmaceutical or nursing profession		5 (11)		16 (16)
Other profession		41 (89)		82 (80)
No statement		0 (0)		5 (5)
School the children attended				
Not yet at school	46 (100)		2 (2)	
Regular school	0 (0)		78 (76)	
School for children with special needs	0 (0)		21 (20)	
Already graduated from school	-		2 (2)	
Own long-term medication (*n* (%))	21 (46)	7 (15)	70 (68)	32 (31)

**Table 2:** Characteristics of children’s long-term medication

**Parameter**	**Children aged 0–5 years (*n*= 46)**	**Children aged 6–17 years (*n*** **= 103)**
Drug formulations of long-term medication n participants (%)		
Liquid peroral dosage form	17 (37)	14 (14)
Solid peroral dosage form	4 (9)	46 (45)
Powder/granules	3 (7)	0 (0)
Injection solutions	2 (4)	17 (17)
Inhaler	2 (4)	6 (6)
Rectal foam/solution	2 (4)	1 (1)
Others	2 (4)	2 (2)

### Experiences of drug-handling problems (part A)

Experiencing drug-handling problems during drug administration was reported by 46 (100%) of parents of 46 children aged 0–5 years, 64 (62%) of 103 children aged 6–17 years, and 72 (70%) of their parents. The described drug-handling problems concerned preparation of the drug, accuracy of dosing, compliance with the time interval, and acceptance of the child as displayed in detail in Tables 3 and 4. In the multivariate analyses of the parent’s group, we found that parents rather reported to experience drug-handling problems (*p* < 0.001) and a higher rate of problems in acceptance (*p* < 0.001) the younger the child was. In the univariate analyses, more parents had experienced problems in drug dosage the younger the child was (*p* = 0.003). This was not significant in the multivariate analysis. In the children’s group, acceptance problems were associated with a lower age (*p* = 0.033) in the univariate analysis, but not in the multivariate setting.

**Table 3:** Concrete drug-handling problems described by all parents (*n* = 46 parents of children aged 0–5 years; *n* = 103 parents of children aged 6–17 years)

**Category of drug-handling problems (*n*** **(%) parents of children aged 0–5 years;** ***n*** **(%) parents of children aged 6–17 years)**	**Examples of concrete problems** **(*n*** **(%) parents of children aged 0–5 years;*n*** **(%) parents of children aged 6–17 years)** **Multiple descriptions per category were possible**	**Examples of parents’ statements**
Preparation of the drug (16 (35%); 25 (24%))	Reconstitution of antibiotic suspensions (7 (15%); 11 (11%))	“The powder clumped.”
		“Extensive foam formation.”
		“Fill mark for water difficult to see due to foam.”
Dosing (28 (61%); 34 (33%))	Difficulties in dosing liquids, especially due to inappropriate dosing devices (26 (57%); 19 (18%))	“Scaling of the dosing syringe not suitable for the volume to be measured.”
		“Dosing spoon is too big for the child’s mouth.”
		“The scale of the measuring cup is not easy to read.”
		“Dosing pipette does not fit to the bottle adapter.”
	Difficulties in dosing tablets, especially in tablet splitting (4 (9%); 14 (14%))	“Tablet without break notch had to be divided for correct dose.”
		“Despite tablet splitter, the tablets are always broken into unequally sized pieces.”
Compliance with the time interval (13 (41%); 51 (50%))	Child was asleep (10 (22%); 23 (22%))	“Child sleeps even though medication should be given.”
		“At night, inhalation and antibiotic administration is difficult, because you always have to wake the child up.”
	Daily routine was not compatible with the time interval for drug intake (4 (9%), 15 (15%))	“Child is at sports.”
		“Child is with another caregiver.”
		“Child has spontaneously been out late in the evening.”
Acceptance (39 (85%); 48 (47%))	Limited acceptance of liquids, especially due to taste or odor issues (37 (80%), 24 (23%))	“Liquids were spat out again.”
		“Liquids were completely refused by pinching the mouth shut.”
		“Liquid could not be administered because it was much too sweet.”
	Decreased acceptance of tablets, especially due to problems in swallowability (3 (7%), 13 (13%))	“Refused due to size and taste.”
		“Child vomited tablet up (too big, too bitter).”
		“Tablet was too big to swallow.”

**Table 4:** Examples of drug-handling problems described by children (*n* = 103 children aged 6–17 years)

**Category of drug-handling problems (*n*** **(%) of children)**	**Examples of children’s statements**
Preparation of the drug formulation (16 (16%))	“Cap of prefilled syringe comes off with difficulty sometimes.”
	“Capsule did not open.”
	“Needle bent while attaching to insulin pen. Belly was slit bloody with needle.”
Dosing (11 (11%))	“The tablet had a division aid, but you couldn’t divide it.”
	“Dosing syringe did not fit.”
	“Dosing spoon was stupid. Bottle opening too big. Everything spilled out.”
	“I didn’t count it right.”
Compliance with the time interval (38 (37%))	“I forgot to take medicine in the evening on a class trip.”
	“In the evening when you go out, it’s sometimes difficult because of food and medication.”
	“Would have liked to eat something, but couldn’t because of the medication.”
	“I was out and did not have the tablets with me.”
	“There was a lot of stress in the morning before school, and then I forgot it.”
Acceptance (32 (31%))	“During the first few years, I didn’t want to inject myself in public.”
	“Syrup doesn’t taste good.”
	“Tablets and capsules difficult to swallow. Now capsules are opened and stirred in.”
	“The tablets were very bitter and came out with the contents of my stomach.”
	“Too big tablets.”
	“Because it hurts.” (note: insulin injections)

### Expectations of the ideal pediatric drug (part B)

#### (B.1) Favored route of drug administration

Of the 46 parents of children aged 0–5 years, 30 (65%) favored a peroral route of drug administration for long-term medication, 8 (17%) preferred the rectal route, 5 (11%) preferred a (trans-)dermal route, and 3 (7%) preferred an injection. The 103 children and their parents stated the following preferences: peroral route (76 (74%) children; 89 (86%) parents), (trans-)dermal route (24 (23%); 13 (13%)), injection (4 (4%); 7 (7%)), rectal route (2 (2%); 1 (1%)), and others ((2 (2%); 2 (2%)). Explanations for preference for the peroral route given by parents were that the peroral route would be the fastest, least painful, and most effective way of drug administration. Children stated they favored the peroral administration because they were used to it (“Drugs go in your mouth”) and it would be the least unpleasant way (“Because anything else feels disgusting to me or would hurt.”).

#### (B.2) Favored characteristics of peroral drugs

When asked if they preferred liquid or solid drug formulations when using peroral drug formulations, 42 (91%) of 46 parents of children aged 0–5 years said they favored liquid drug formulations, and 3 (7%) preferred solid drug formulations. Of 103 children aged 6–17 years and their parents, 42 (41%) children and 47 (46%) parents preferred liquids; 38 (37%) children and 41 (40%) parents favored solid dosage forms. In multivariate analysis of the parent’s group, the liquid dosage form was more favored the younger the child was (*p* = 0.003). In the multivariate analysis of the children’s group, intake of long-term medication by the child was a predictor for the preference of solid dosage forms (*p* = 0.005).

The most important characteristics of solid and liquid drug formulations (e.g., size, color) are shown in Table 5. The children’s choice of peroral drug formulations and of the most important characteristics of solids and liquids were independent of age (n.s.). Significant age differences were only found for the flavor of peroral drug formulations. While children who preferred sweet flavors had a median age of 10 years (Q25/Q75: 8/14, min/max: 6/17), the median age of children who preferred neutral flavors was 14 years (Q25/Q75: 11/16, min/max: 6/17; *p* = 0.01). In the univariate analysis of the parents’ group, sweet flavors were preferred for younger children (0.033). This was also found in the multivariate analysis of the children’s group (*p* = 0.001).

**Table 5:** Favored characteristics of peroral drug formulations according to participants’ answers to a questionnaire with pre-set answers to tick. Multiple answers were not intended, but if participants were not able to choose only one answer, all chosen answers were considered for the evaluation

	**Parents of children aged 0–5 years (*n* = 46)**	**Children aged 6–17 years (*n* = 103)**	**Parents of children aged 6–17 years (*n* = 103)**
Preference of solid or liquid peroral drug formulations, *n* participants (%)			
Liquid	42 (91)	42 (41)	47 (46)
Solid	3 (7)	38 (37)	41 (40)
Does not matter	0 (0)	23 (22)	15 (15)
Others	1 (2)	0 (0)	0 (0)
Most important characteristic of solid peroral drug formulations, *n* participants (%)			
Size	29 (63)	58 (56)	83 (81)
Flavor	7 (15)	37 (36)	25 (24)
Color	5 (11)	12 (12)	7 (7)
Shape	2 (4)	12 (12)	13 (13)
Label on the surface	3 (7)	2 (2)	4 (4)
No answer	0 (0)	1 (1)	0 (0)
Most important characteristic of liquid peroral drug formulations, *n* participants (%)			
Volume	15 (33)	21 (20)	17 (17)
Flavor	27 (59)	73 (71)	76 (74)
Color	0 (0)	4 (4)	4 (4)
Texture	1 (2)	6 (6)	7 (7)
Smell	3 (7)	16 (16)	30 (29)
Preferred flavor of drug, *n* participants (%)			
Sweet	24 (52)	55 (53)	46 (45)
Neutral	21 (46)	39 (38)	60 (58)
Bitter	1 (2)	0 (0)	0 (0)
Does not matter	0 (0)	9 (9)	1 (1)

### Comparison of children’s and parents’ preferences concerning route of drug administration and favored characteristics of peroral drugs

Regarding the favored route of drug administration, parents’ assessment matched with their children’s answer in 68/103 (66%) cases. Answers concerning the preference for solid or liquid drug formulation matched in 62/103 (60%) cases; matches regarding the other characteristics were as follows: flavor 61/103 (59%), most important characteristic of solid peroral drug 48/103 (47%), and most important characteristic of liquid peroral drug 44/103 (43%). The matching of the parent’s and the child’s answers was independent of the child’s age (n.s.) in all categories.

#### (B.3) Description of the ideal pediatric drug in their own words

Answers to an open question regarding the ideal pediatric drug formulation were highly heterogeneous. Examples are shown in Table 6.

**Table 6:**
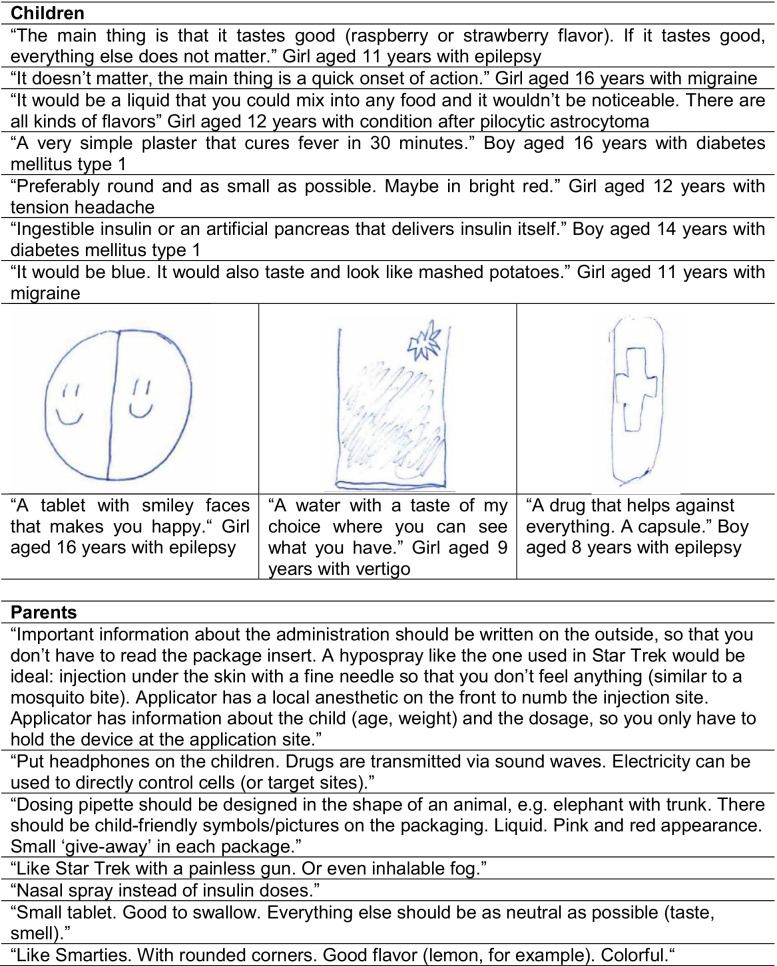
Examples for specific expectations of parents and children regarding the ideal pediatric drug

## Discussion

Data of literature [8] and our own clinical experience show that the needs of children and their parents should be more carefully considered in practical drug administration. Therefore, we explored the main causes of problems in drug handling by children and their parents. The ideas of children and their parents about how a medicine should best be administered were of particular interest in this survey. For this purpose, we performed a prospective observational study on children’s and their parents’ experiences of drug administration and their expectations of the ideal pediatric drug. A high proportion of children and parents reported having experienced drug-handling problems. Concerning preferred characteristics of an ideal pediatric drug, most answers were highly heterogeneous and often differed between children and their parents.

### Experiences of drug-handling problems (part A)

A high proportion of children and parents reported previous drug-handling problems in the context of pediatric drug therapy. The described problems were in the context of preparation of the drug formulation, dosing accuracy, compliance with the time interval between two administrations, and the children’s acceptance of the drug.

Many parents reported problems with the preparation of oral antibiotic suspensions. They described, for example, that the powder clumped, that they experienced extensive foam formation, and that the fill mark for water was difficult to see due to foam. Since antibiotics are almost exclusively used for acute treatment, parents are usually not familiar with their handling. In an earlier study, a pharmacist’s verbal education supported by photographic education material was much more effective in providing information on correct preparation of oral antibiotic suspensions to caregivers than the sole provision of package leaflets or education sheets [9].

A high proportion of parents reported problems in dosing liquids, especially due to dosing devices they experienced as inappropriate. This is in line with data from literature that show that more than 40% of parents made dosing errors when preparing liquid medications. With advanced counseling and provision of dosing aids, those errors could be reduced [10]. However, different dosing aids show different accuracy. In particular, medicine cups that parents like to use have a reduced accuracy [11, 12]. Thus, parents should be educated on the correct use of devices with a higher accuracy such as syringes. Besides, the examples provided by the parents in our study show that many devices are inappropriate with scales difficult to read, spoons too big for the child’s mouth, or dosing pipettes not fitting to the bottle adapter. Those problems have to be addressed by pharmaceutical companies.

The participants of our study also described problems concerning the splitting of tablets. This is in accordance with data from literature. For example, it was shown that in spite of functional break lines, the quartering of 10 mg hydrocortisone tablets caused unacceptable dose variations. Thus, the authors of the study favor mini-tablets in adequate dosages for children [13].

Participants of our study also reported difficulties in adherence to the time intervals for medication intake due to periods of sleep or missing compatibility of the intervals with daily routines. To increase adherence as well as quality of life, physicians, parents, and their children should aim at finding time intervals that are compatible with the families’ daily lives.

The participants of our study described reduced acceptance of medicines due to taste or odor issues or difficulties in swallowability. The preferences of children concerning those issues should be considered. Children’s preferences about their medication are very individual. This, however, makes it difficult to achieve the goals that have been set to improve drug handling in this population.

To ensure the safety and effectiveness of a drug therapy, drug-handling problems should be addressed and, whenever possible, prevented. Studies have shown that pharmacist intervention reduces medication errors in drug administration [14–17]. The instructions should be explained in a precise, simple, and understandable way. Placebo medicinal products and pictograms can increase comprehensibility. Children’s adherence can also be improved if they are actively involved in their medication process as early as possible [18].

### Expectations of the ideal pediatric drug (part B)

The peroral route of drug administration was preferred by most participants. This is in accordance with earlier studies that describe barriers to administering non-peroral formulations to children [19]. Thus, in drug development and prescription, the focus should be on peroral drug formulations.

The children’s favored characteristics of drug formulations were highly individual. Only when asked about the favored taste of peroral dosage forms, an age-dependent effect could be shown. Sweet flavors were preferred by younger children while older children tended to prefer neutral flavors. This is in accordance with findings of earlier studies that infants and children showed an elevated preference for sweet flavors [20]. Considering the individual expectations of children and adolescents, ideally different formulations of an active ingredient should be available to meet the children’s preferences as much as possible. Through an increased collaboration between physicians, pharmacists, and parents and their children, the choice of the most suitable drug formulation can be supported [21]. Even if not every administration problem leads to severe clinical consequences, it should be considered that drug-handling problems are closely related to a negative impact on adherence [22]. Therefore, resolving drug-handling problems can not only eliminate direct consequences of the problems themselves, but also reduce indirect consequences of decreased adherence. In the present study, we aimed at identifying problems from the perspectives of children and their parents and to elaborate their proposed solutions for future activities.

Preferences of children and parents concerning drug formulations differed in around half of the reports. Our findings are in line with earlier studies that have shown that children are indeed well able to express experiences and expectations concerning their drug therapy [23, 24]. Nevertheless, physicians tend to talk to the parents when they aim at exploring the children’s needs. As each child, however, has individual desires and needs, pediatricians should communicate directly with children about their drug therapy and disease management at an appropriate cognitive level. While it takes more time and empathy compared to the communication with parents, children have sophisticated information needs concerning health issues [25, 26]. In addition, when it comes to the appropriate drug formulation, children need to be actively involved in the decision-making process to reach optimal acceptance and adherence with the prescribed drug therapy. As we could show, parents were not always able to properly assess their children’s needs. For example, the preference of liquid or solid peroral drugs depended in the children on whether the child took long-term medication but this was not reflected in the parents’ preference.

A particularly interesting finding of our study was single reports on proposed ideal drugs from children and parents. The suggested dosage forms ranged from the smallest possible tablets, to transdermal systems, to nasal spray, or even to mist and to utopian transmission by means of (sound) waves via headphones. The onset of action should be fast and the ideal taste ranges from neutral to pleasantly flavored, e.g., lemon or strawberry, up to savory flavors like mashed potatoes or pizza. Free choice of taste and the possibility of mixing the medicine into food were also preferred as far as taste was concerned. Red color, animal shapes, and smiley faces were mentioned visual preferences on tablets, particularly but not only mentioned by children. Parents would like to see more easy-to-read instructions for use on the package or an included anesthesia for auto-injectors.

In summary, we found a high variability in preferences that are reflected in the responses to the open question about the ideal pediatric drug. The answers were very individual and did not reveal a clear pattern. The children and their parents addressed not only practical aspects such as instructions for drug handling printed on the package, but also futuristic technological drug formulations such as the communication of effects via headphones.

#### Limitations

We were only able to directly address the perspective of children who had the maturity and intellectual capacity to answer the questions. In the case of younger children, only the parents’ perspective could be directly explored. Due to the explorative character of this study aiming at investigating concise problems pediatric patients and their parents face in everyday drug handling, we did not measure patient-related outcomes such as drug adherence.

## Conclusion

Most pediatric patients and their parents had already experienced drug-handling problems. Expectations concerning the ideal pediatric drug formulation were highly individual and often differed between children and their parents. Thus, to improve pediatric drug therapy, the individual expectations of the patients should be considered and children should be approached directly to get to know their perspective as exactly as possible as far as they have the maturity and intellectual capacity to express their wishes.

## Supplementary information

Below is the link to the electronic supplementary material.

Supplementary file1 (PDF 130 KB)

## Data Availability

The data presented in this study are available on request from the corresponding author.
