# Supplementary material for: Drug-handling problems and expectations of the ideal pediatric drug—reported by children and their parents
Source: Eur J Pediatr. 2022 Feb 23;181(5):2161–71. doi: 10.1007/s00431-022-04419-6 (PMC9056485; doi:10.1007/s00431-022-04419-6)
Supplement: Supplementary file 1 — Supplementary file1 (PDF 130 KB) [file 431_2022_4419_MOESM1_ESM.pdf]

Supplement 1

1 Questionnaire for parents and caregivers of children aged 0 to 17 years

Information about your child

The following questions apply only to your child who is being treated in the children's hospital today.

Age

__________ years

Sex

☐ male ☐ female ☐ other

Which school type does your child attend?

☐ regular school
☐ school for children with special needs
☐ not yet at school
☐ already graduated from school

Does your child need to take medication due to a chronic disease? *Multiple answers possible*

☐ yes, regularly (e.g. every day)
☐ yes, in an emergency situation
☐ no
☐ don't know

If yes, what medications does your child receive?

**Name of the medicine**	**Dosage form** *(e.g. liquid, tablets, capsules, suppositories, drops, ...)*	**Dosing scheme** *(morning - noon - evening - at night) or if necessary*

1 Have you ever had difficulties in the past ...

1.1 ... to prepare your child's medication correctly?

Example: *Problems in the preparation of oral suspensions, capsules could not be opened, protective cap did not open, ...*

□ yes: ____________________
□ no
□ don't know

1.2 ... to measure your child's medication correctly?

Example: *Dosing pipette did not fit on the bottle, tablets could not be divided, ...*

□ yes: ____________________
□ no
□ don't know

1.3 ... to adhere to the time interval for administering your child's medication?

Example: *Child slept, child was not present, ...*

□ yes: ____________________
□ no
□ don't know

1.4 ... with your child's acceptance when administering the medication?

Example: *Child fought back, child spat out everything, ...*

□ yes: ____________________
□ no
□ don't know

2 What is your favored route of drug administration for your child's long-term medication?

☐ 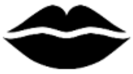
peroral

☐ 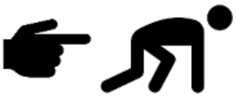
rectal

☐ 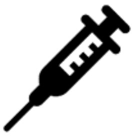
through the skin *(e.g. injections)*

☐ 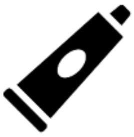
(trans-) dermal *(e.g. cream / patch)*

☐ Other: ____________________

2.1 Why is this your preferred route of drug administration?

3 Do you prefer solid or liquid peroral drug formulations for your child?

□ solid peroral drug formulations *(e.g. tablets or capsules)*

□ liquid peroral drug formulations *(e.g. drops or oral suspensions)*

□ doesn't matter

□ other: ___________________________

4 How should a peroral drug taste for your child?

□ bitter

□ neutral

□ sweet

□ doesn't matter

□ other: __________________________

5 What is the most important characteristic of solid peroral drug formulations for the child's acceptance of a medicine?

□ size
□ shape *(e.g. round or square)*
□ color
□ flavor
□ label on the surface *(e.g. symbol on the tablet)*

6 What is the most important characteristic of liquid peroral drug formulations for the child's acceptance of a medicine?

□ smell
□ color
□ flavor
□ texture *(e.g. how viscous the suspension is, or whether particles float in the liquid)*
□ volume

7 If you could invent a drug for your child, what would it be like?

Information about yourself

Age

__________ years

Sex

□ male □ female □ other

What is the highest level of your professional education? What profession do you currently have?

Professional education: □ no degree □ vocational □ university degree

Current profession: □ medical, pharmaceutical or nursing profession □ other profession

Do you take medication yourself due to a chronic illness?

□ yes, regularly (e.g. daily or weekly)
□ yes, on demand (e.g. in emergency situations)
□ no

2 Questionnaire for children aged 6 to 17 years

1 Have you ever had difficulties in the past to ...

1.1 ... prepare the medication correctly?

Example: *The capsule could not be opened or the oral suspension leaked*

□ yes: ______________________________

□ no

□ don't know

1.2 ... measure the medication correctly?

Example: *The tablet could not be divided or the dosing syringe did not fit on the bottle*

□ yes: ______________________________

□ no

□ don't know

1.3 ... adhere to the time interval for taking medication?

Example: *You were not at home and you did not have your medication with you*

□ yes: ______________________________

□ no

□ don't know

1.4 ... take the medication correctly?

Example: *The drug did not taste good and you spat out everything*

□ yes: ______________________________

□ no

□ don't know

2 Imagine you have to take a medication every day. Which route would you prefer?

□

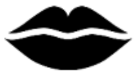

via the mouth

□

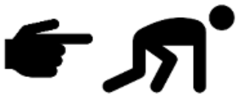

via the rectum

□

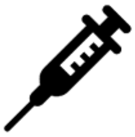

through the skin *(e.g. injections)*

□

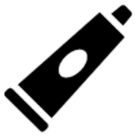

on the skin *(e.g. cream / patch)*

□

other:

____

2.1 Why do you think that's the best?

3 Would you rather take solid or liquid peroral drug formulations?

- □ solid peroral drug formulations *(e.g. tablets or capsules)* [pictures provided of several solid peroral drug formulations]
- □ liquid peroral drug formulations *(e.g. drops or suspensions)* [pictures provided of several liquid peroral drug formulations]
- □ doesn't matter
- □ other: ________________________

4 How should your peroral medicine taste?

- □ bitter
- □ neutral
- □ sweet
- □ doesn't matter
- □ other: ________________________

5 What is the most important characteristic of solid peroral drug formulations for you?

- □ size
- □ shape *(e.g. round or square)*
- □ color
- □ flavor
- □ label on the surface *(e.g. symbol on the tablet)*

6 What is the most important characteristic of liquid peroral drug formulations for you?

- □ smell
- □ color
- □ flavor
- □ texture *(e.g. how viscous the suspension is, or whether particles float in the liquid)*
- □ volume

7 If you could invent a drug for children, what would it be like? Can you describe it?
